# Supplementary material for: Taste shaped the use of botanical drugs
Source: eLife. 2024 Jan 24;12:RP90070. doi: 10.7554/eLife.90070 (PMC10945733; doi:10.7554/eLife.90070)
Supplement: Supplementary file 3. [file elife-90070-supp3.docx]

**Supplementary file 3. Codes and the short descriptions of the 22 chemosensory qualities to represent tastes and flavours. Codes are those used in Supplementary files 2,5 and 6; short descriptions match those used in Figure 1.**

1. **AROM**: Aromatic

2. **ASTR**: Astringent

3. **BALS**: Balsamic

4. **BITT**: Bitter

5. **BURN**: Burning/Hot

6. **FRES:** Fresh/Cooling

7. **FRUI:** Fruity

8. **HERB**: Herby/Leafy

9. **MUCI:** Mucilaginous

10. **MUSK**: Musky

11. **NUTT**: Nutty

12. **SALT**: Salty

13. **SMOK**: Smoky

14. **SOAP**: Soapy

15. **SOUR**: Sour/Acidic

16. **STAR**: Starchy

17. **STGI**: Stinging/Mustardy

18. **STNK**: Stinky

19. **STRW**: Straw-like

20. **SWEE**: Sweet

21. **WARM**: Warming

22. **WOOD**: Woody
